# Stress-Induced Changes in Nucleocytoplasmic Localization of Crucial Factors in Gene Expression Regulation

**DOI:** 10.3390/ijms25073895

**Published:** 2024-03-31

**Authors:** Ali Khamit, Payal Chakraborty, Szabolcs Zahorán, Zoltán Villányi, Hajnalka Orvos, Edit Hermesz

**Affiliations:** 1 Department of Biochemistry and Molecular Biology, Faculty of Science and Informatics, University of Szeged, H-6701 Szeged, Hungary; ali.khmt@gmail.com (A.K.); payal96.pharm@gmail.com (P.C.); szabolcs.zahoran@unige.ch (S.Z.); villanyi22@gmail.com (Z.V.); 2 Department of Obstetrics and Gynecology, Albert Szent-Györgyi Medical School, University of Szeged, H-6701 Szeged, Hungary; kokutine.orvos.hajnalka@med.u-szeged.hu

**Keywords:** blood vessels, CNOT1 (carbon catabolite repression-negative on TATA-less), fetal development, maternal smoking, PARP-1 (poly-(ADP-ribose) polymerase-1), rescue mechanism, RPB1 (the largest subunit of RNA polymerase II enzyme)

## Abstract

This study investigates the toxic effect of harmful materials, unfiltered by the placenta, on neonatal umbilical cord (UC) vessels, focusing on stress-induced adaptations in transcriptional and translational processes. It aims to analyze changes in pathways related to mRNA condensate formation, transcriptional regulation, and DNA damage response under maternal smoking-induced stress. UC vessels from neonates born to smoking (Sm) and nonsmoking mothers (Ctr) were examined. Immunofluorescence staining and confocal microscopy assessed the localization of key markers, including Transcription Complex Subunit 1 (CNOT1) and the largest subunit of RNA polymerase II enzyme (RPB1). Additionally, markers of DNA damage response, such as Poly(ADP-ribose) polymerase-1, were evaluated. In Sm samples, dissolution of CNOT1 granules in UC vessels was observed, potentially aiding stalled translation and enhancing transcription via RPB1 assembly and translocation. Control vessels showed predominant cytoplasmic RPB1 localization. Despite adaptive responses, Sm endothelial cells exhibited significant damage, indicated by markers like Poly(ADP-ribose) polymerase-1. Ex vivo metal treatment on control vessels mirrored Sm sample alterations, emphasizing marker roles in cell survival under toxic exposure. Maternal smoking induces specific molecular adaptations in UC vessels, affecting mRNA condensate formation, transcriptional regulation, and DNA damage response pathways. Understanding these intricate molecular mechanisms could inform interventions to improve neonatal health outcomes and mitigate adverse effects of toxic exposure during pregnancy.

## 1. Introduction

Smoking is recognized as one of the risks and determining factors for the formation of endothelial dysfunction in adulthood. More than 4800 substances are present in the particulate and vapor phases of cigarette smoke, including free radicals and toxic metals. Metals play a crucial role in the pathophysiological mechanisms of nearly all smoking-caused diseases: cancers, retinal degeneration, chronic obstructive pulmonary diseases, renal dysfunctions, cardiovascular diseases, neurological disorders, degeneration of bone, immune dysfunctions, and problems during embryonic development. The developmental effects of cadmium (Cd) and arsenic, the best studied metals from cigarette smoke, are often manifested in vivo as reduced fetal weight, malformations, and altered neurobehavioral development [1]. In addition to their impact on growth parameters, they are established epigenetic modifiers; thus, there is the potential that prenatal exposure to the above-mentioned metals may also have long-term implications on child development [2]. The elevated level of pro-oxidant compounds highly disturbs the redox homeostasis conditions. Consequently, functional capacities of proteins, nucleic acids, and lipids get deprived, which alters the regulatory cascades [3]. Similarly, during embryonic and fetal development, there arises a significant threat to the vascular system due to oxidative/nitrosative stress, which directly or indirectly gets triggered by the toxic components originated from maternal smoking. Toxic materials and reactive oxygen species (ROS) easily traverse through the placenta and potentially disrupt the carefully orchestrated molecular mechanisms that highly promote the dynamic functioning of regulatory systems and gene expression pathways [4,5]. One of the primary targets for any unfiltered harmful substances is the endothelial cell population of the umbilical cord (UC) vessels. Since the fetal vascular system can be considered the elongation of the UC vessels, any kind of structural and functional alterations in the vessels can be an utmost determining factor for fetal development or even for later lifetime. In addition to the UC vascular system, the circulating fetal red blood cells (RBCs) are also directly affected by the unfiltered toxic materials and become a source of reactive oxygen and nitrogen species [6]. As we hypothesized and proved in our latest publications, there is an active communication between the vessel endothelial cells and RBC population; thus, changes in the structure and molecular network of any affect the functionality of the other [7,8].

In this study, we have picked three pathways to study under maternal smoking-induced stress. Two of these are parts of eukaryotic gene regulation, including (i) transcription, and (ii) formation of cytoplasmic mRNA condensates coupled with either degradation of mature cytoplasmic mRNA or with protein complex assembly, and one surveillance pathway of (iii) DNA damage response.

First, we focused on one of the multifaceted key nuclear sensors of DNA damage, the poly-(ADP-ribose) polymerase-1 (PARP-1) enzyme. PARP-1 is among the initial proteins that accumulate to the damage sites through its DNA binding domain and its activation to synthesize poly-ADP-ribose [9,10]. The covalent modification of specific proteins (such as H1 histone) by poly-ADP-ribosylation induces chromatin relaxation and facilitates repair enzymes recruitments to the damaged DNA. Apart from its primary activity in the routine repair of DNA damage, PARP-1 can also be associated with the diverse physiological and pathological functions, such as transcription, cell survival, and several forms of cell death. PARP-1 interacts with a wide array of cell death proteases [11,12,13,14]. In case of severe DNA damage, PARP-1 is cleaved by caspase-3 (Casp3) within the nuclear localization signal near the DNA-binding domain, and as a result the cleaved PARP-1 fragment is considered to be a distinctive indicator for apoptosis [15,16]. To gain valuable insights into the extent of DNA damage that blood vessel endothelial cells endure under stress conditions induced by smoking, it is crucial to examine the nucleocytoplasmic localization of PARP-1, both in its cleaved and full-length forms. This approach provides significant data, allowing a comprehensive assessment of the severity of DNA damage in these cells.

Oxidative stress affects gene expression at all levels of regulation. Adaption to stressful conditions requires prompt responses from the transcriptional and translational machineries of the cells. The formation and maintenance of a balanced protein profile for cell survival is orchestrated by complex systems that coordinate nuclear and cytoplasmic processes [17]. In this context, we followed the cytoplasmic and nuclear localization of RPB1, the largest subunit of RNA polymerase II enzyme. RPB1 serves as a platform for assembly of factors that regulate transcriptional machinery. RPB1 has a crucial role not only in the assembly of the RNA polymerase II complex but even in the carefully coordinated nucleocytoplasmic shuttling of the complex [18]. RPB1 is conserved among eukaryotes with various numbers of C-terminal heptapeptide repeats, which makes it vulnerable for frequent cytoplasmic aggregation [19]. This latter phenomenon can occur co-translationally, therefore suitable to monitor translational accuracy and chaperoning efficacy of the cell [20]. Some of the protein factors escort mRNAs from the nucleus to the cytoplasm and provide quantitative and qualitative control of protein synthesis on the ribosomes. These bidirectional coupling of nuclear and cytoplasmic processes enable the cells to rapid adaptation. Transcription Complex Subunit 1 (CNOT1), a key component of the complex Carbon Catabolite Repression-Negative on TATA-less (Ccr4-Not) complex, plays a crucial role in regulating mRNA metabolism and gene expression through its involvement in mRNA deadenylation and degradation processes [21]. CNOT1 acts as a scaffold protein, coordinating the assembly of various factors involved in mRNA decay and translation repression and—as recently discovered—in cytoplasmic mRNA condensate formation [19,20,21,22]. Additionally, CNOT1 has been implicated in diverse cellular processes, including stress response and embryonic development. The morphological and molecular changes in the endothelial cell population of the UC vessels may impair normal cell function during embryonic/fetal development. In our earlier studies, we presented evidence on damaged cell structure and altered gene expression profile in the endothelial layer of UC originated from neonates born to a smoking mother. Based on the ultrastructure of UC vessels, samples with a smoking origin showed significant differences. Many of the endothelial cells lose their intercellular contacts and exhibit a “dying” phenotype, most likely due to long-term adverse conditions. The common phenotypical features of these samples were the nuclear fragmentation, cytoplasmic shrinkage, detachment from basement membrane formation of huge inter- and intracellular vacuoles, and significant increase in the apoptotic cell population. Veins were always affected to a greater extent, probably due to their primary exposition to toxic materials, unfiltered by the placenta [23]. Along with the morphological changes, altered gene expression profiles were also detected with significant correlation to damage of red blood cell populations [8].

In this study, we investigated the molecular background of altered protein profile, focusing mostly on the nucleocytoplasmic localization of specific regulators of transcriptional/translational machineries, such as PARP-1, the DNA damage sensor in the nucleus, RPB1 the largest subunit of RNA polymerase II enzyme, and CNOT1 as the key player in quality control of several gene expression levels.

## 2. Results

### 2.1. PARP-1 Nuclear Localization and Cleavage Is Carried out in a Stress-Dependent Manner in the Endothelial Cell Population

**Figure 1:**
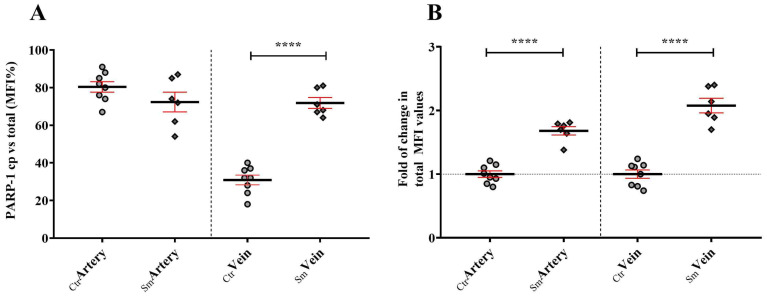
Cytoplasmic localization (**A**) and expression level (**B**) of PARP-1 among control (n_Ctr_ = 8) and smoking origin derived umbilical cord vessels (n_Sm_ = 6). Dot plots represent the semiquantified results of PARP-1 specific immunolabeling on the arterial and venous endothelial cells. Dots symbolize the overall MFI values in percentiles of the cytoplasmic distribution from individual samples (**A**). Fold of change in total MFI values (**B**) where summarized data from the control group were applied as the baseline of comparison (mean ± SEM). Statistics: unpaired *t*-test (two-tailed) followed by Mann-Whitney test to compare ranks (**** *p* ≤ 0.0001).

As is well documented, cigarette-smoke-originated ROS induces genome instability because of DNA damage. PARP-1 is generally thought to be compartmentalized in the nucleus, where it takes part in the surveillance of genome integrity, including repair mechanisms. Here, we analyzed the nuclear and cytoplasmic localization of the enzyme by immunolabeling with PARP-1-specific antibody (tPARP-1). The signal was predominantly restricted to the cytoplasm both in the control (Ctr) and the Sm arterial endothelial cells, ~80% and ~70% of the mean fluorescent intensity (MFI) value, respectively. In the Ctr vein, PARP-1 labeling was detected mostly in the nucleus, ~70% of the MFI values, while in the Sm group, the majority of signal, ~70%, was measured in the cytoplasm (Figure 1A and Supplementary Figure S1). The MFI values for PARP-1 was always higher in samples with smoking background than in the controls; an ~1.5-fold difference was measured in the arteries and an ~1.7-fold in the case of the veins (Figure 1B).

**Figure 2:**
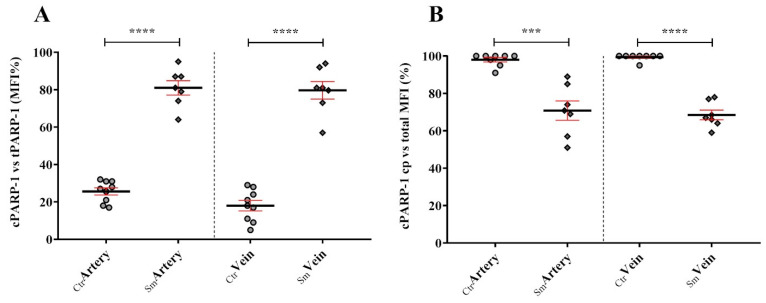
Percentile of fragmented versus total PARP-1 (cPARP-1/tPARP-1) MFI values (**A**) and the overall cPARP-1 MFI in percentiles of the cytoplasmic distribution (**B**). Dot plots represent the semiquantified results of tPARP-1 and cPARP-1 specific immunolabeling on the arterial and venous endothelial cells among control (n_Ctr_ = 8) and smoking origin derived umbilical cord vessels (n_Sm_ = 6). Statistics: unpaired *t*-test (two-tailed) followed by Mann-Whitney test to compare ranks (*** *p* ≤ 0.001 and **** *p* ≤ 0.0001).

While PARP-1 is a key nuclear sensor of DNA damage, the cleaved 89 kDa C-terminus fragment of it (cPARP-1) might serve as a specific marker for apoptosis. Along with tPARP-1 antibody, which is able to identify both the full length and the cleaved PARP-1 fragments, a cPARP-1 specific antibody was used, which was raised against a short amino acid sequence containing a neoepitope near the C-terminal cleavage site. In the control arteries, cPARP-1 was exclusively localized to the cytoplasm, and the intensity was ~25% of the cytoplasmic total PARP-1. In the case of the Sm sample populations, cPARP-1 was mostly paralleled with the total PARP-1 pattern, even in the nucleus, having ~80% intensity of the cytoplasmic total PARP-1. In the control veins, cPARP-1 was localized to the nucleus with a much lower intensity than measured with tPARP-1 antibody (~20%). In the case of smoking-related samples, cPARP-1 gets fully co-localized with total PARP-1, showing ~80% intensity similarity (Figure 2A,B).

**Figure 3:**
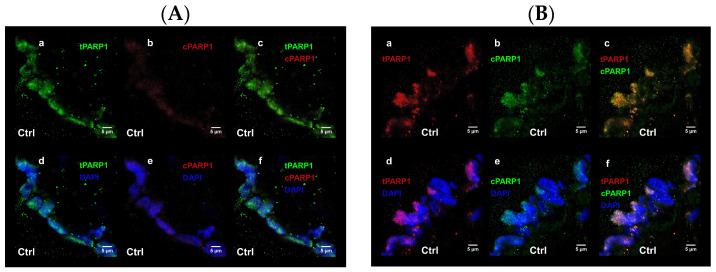
Representative confocal images were captured on total and fragmented PARP-1 double immunolabeled control veins, without (**A**) and with (**B**) 0.5 ng/µL Cd^2+^ exposure. For immunolabeling monoclonal rabbit anti-tPARP-1 (**a**,**d**) and mouse anti-cPARP-1 (**b**,**e**), antibodies with 1:100 dilution were used, followed by Alexa 488 (green) and Alexa 647 (red) anti-rabbit/anti-mouse secondary antibodies labelling with dilution 1:1000. Cell nuclei were counterstained with 1 µg/mL 4′, 6-diamidino-2-phenylindole (blue). Panels (**c**,**f**) present the merged images. Slides were mounted and examined using a confocal laser scanning microscope, ZEISS LSM 880 equipped with Axiocam 503 mono.

To connect the increased PARP-1 cleavage to smoking habit, an ex vivo Cd^2+^ treatment was set up and followed the cleavage frequency in the vein of Ctr UC fragments. The cPARP-1 pattern was similar to total PARP-1 localization with ~80% intensity homology (Figure 3A,B). Short, high doses of Cd^2+^ treatment induced immediate fragmentation of tPARP-1, similar to the effect of maternal smoking.

### 2.2. Caspase3 Localization in the Endothelial Cell Population Is Stress Dependent

Casp3, as one of the executioner caspases, migrates into the nucleus by active transport mechanism and induces cell death. PARP-1 is one of the cellular substrates of Casp3 for cleavage, and the resulting fragment is also a predictive hallmark for the caspase-dependent apoptosis. Nuclear accumulation of the active Casp3 was examined by immunolabeling using a cleaved-Casp3 specific antibody.

**Figure 4:**
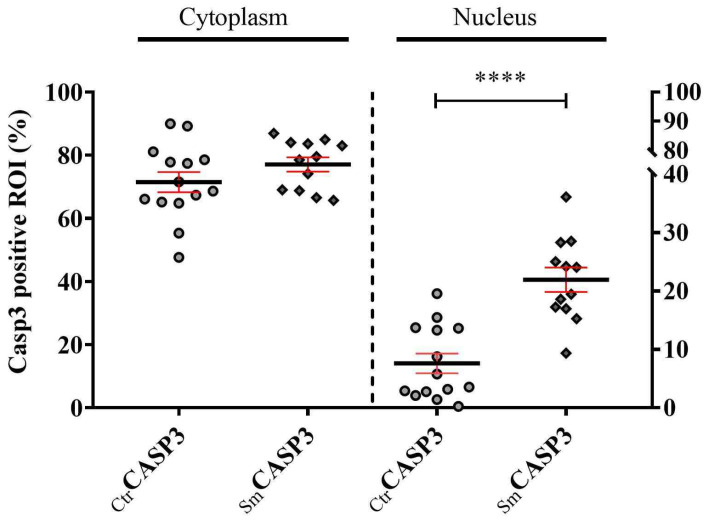
Cytoplasmic and nuclear distribution of cleaved Casp3. Casp3 localization was shown in the venous endothelial cells of umbilical cord, taking n_Ctr_ = 14 and n_Sm_ = 12 individual samples immunolabeled with antibody specific for cleaved Casp3. Statistical analysis was done by using unpaired *t*-test (two-tailed) and Mann-Whitney post hoc test to compare ranks (**** *p* ≤ 0.0001).

The localization of the active Casp3 showed a stress-dependent pattern. In the control population, both in the arteries and the veins Casp3 was mostly localized to the cytoplasm. In the Sm group, a significant increase was detected in the active Casp3 level, where the cytoplasmic accumulation was like the control population in both vessel types; however, in the nuclei a nearly threefold elevation was detected in the vein compared to the Ctr group (Figure 4), indicating cellular commitment toward apoptosis.

### 2.3. Immunodetection of CNOT1 and RPB1 in the UC Vessels

The CCR4-NOT complex, involved in a variety of distinct gene expression related processes, is present in both the cytoplasm and the nucleus of eukaryotic cells. The localization of CNOT1, the largest subunit in the complex, was followed by double immunolabeling on the UC sections using antibodies specific against CNOT1 and the RNA polymerase II large subunit, RPB1.

**Figure 5:**
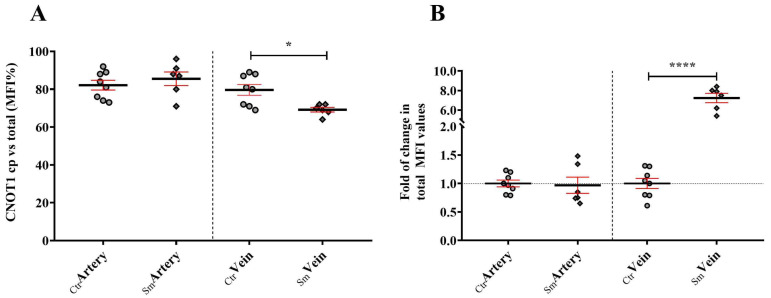
Cytoplasmic localization (**A**) and expression level (**B**) of CNOT1 among control (n_Ctr_ = 8) and smoking origin derived (n_Sm_ = 6) umbilical cord vessels. Dot plots showed the semiquantified results of CNOT1 specific immunolabeling on the arterial and venous endothelial cells. Dots and rhombuses (**A**) symbolize the overall MFI values in percentile for the cytoplasmic distribution of individual samples. Fold of changes in the total MFI values (**B**), where the summarized data from control group were applied as the baseline of comparison (mean ± SEM). Statistics: unpaired *t*-test (two-tailed) followed by Mann-Whitney test to compare ranks (* *p* ≤ 0.05, **** *p* ≤ 0.0001).

A large extent of CNOT1 was present in the cytoplasm regardless of the sample origin and the vessel type; in the Ctr arteries 75–90% and the vein ~85%, whereas in the Sm arteries 90% and the vein 70% of the total MFI values (Figure 5A and Supplementary Figures S2 and S3b,c). In addition, CNOT1 in the cytoplasm was found to be in the form of discrete foci in both vessel types of the control samples. However, in the Sm population, the CNOT1 signal showed a more diffused pattern and fewer distinct foci were observed, and in parallel an overall ~7-fold increase in MFI in the veins compared to the control (Figure 5B).

**Figure 6:**
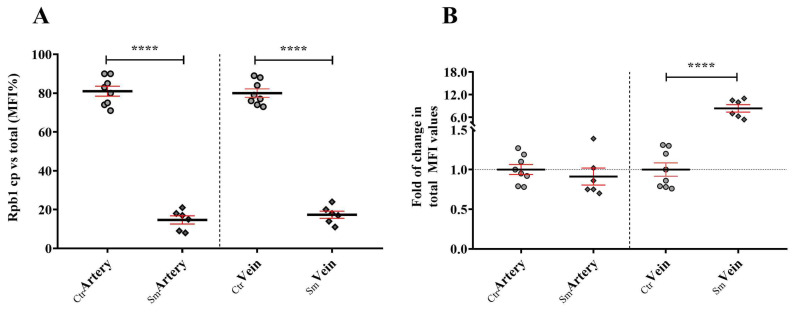
Cytoplasmic domination (**A**) and expression level (**B**) of RPB1 among control (n_Ctr_ = 8) and smoking origin derived (n_Sm_ = 6) umbilical cord vessels. Dot plots present the semiquantified results of PARP-1 specific immunolabeling on the arterial and venous endothelial cells. Dots and rhombuses (**A**) symbolize the overall MFI values for the cytoplasmic distribution in the percentile form, as derived from the individual samples. Fold of changes in the total MFI values (**B**), where the summarized data from the control group were applied as the baseline of comparison (mean ± SEM). Statistics: unpaired *t*-test (two-tailed) followed by Mann-Whitney test to compare ranks (**** *p* ≤ 0.0001).

In contrast, we observed a marked significant difference in the RPB1 localization between the two sample groups. In the control UC vessels, it accumulated mostly in the cytoplasm (75–80%), whereas in the Sm UCs significant nucleoplasmic localization was detected both in the arteries and in veins, ~80–90% of the total MFI value, depending on the sample population (Figure 6A). Additionally, we did not observe a typical pattern of cytoplasmic aggregation of RPB1 neither in the Sm nor in the control samples, but a 6–11-fold increase in the MFI value was measured in the Sm veins compared to control samples (Figure 6B, and Supplementary Figures S2 and S3a,d–f).

## 3. Discussion

Maternal smoking induced morphological and molecular alterations in endothelial cells of UC vessels might be linked to pathophysiological mechanisms, leading to neonatal cardiovascular disorders. In our previous studies, we have clearly demonstrated that damage in the vessels is proportional to the level of exposure. Accordingly, the primarily exposed vein was the most affected at all levels, but significant alterations were also detected in the arterial endothelium, designating an overall, extensive functional impairment [23]. Since the exposure level of the circulated RBCs comparable to that of the veins and the continuous contact with the endothelial layer of the blood, the comprehensive destruction of the vascular system is not surprising.

The work presented in this paper focused mainly on cell survival and activation of stress-response pathways through granule formation, using sample sets, tested before by molecular and structural studies [23]. The main findings of the present study are as follows: (a) PARP-1 is mostly localized into the cytoplasm of the UC vessel’s endothelial cells regardless of the sample origin. However, in the Sm population a significant part of the enzyme is already inactivated. The fragmentation of the enzyme in this group can be connected to the toxic metal exposure (among others) due to maternal smoking and can be mimicked with Cd^2+^ treatment of the Ctr samples. (b) Furthermore, CNOT1 gets mostly linked to the cytoplasm in both the Ctr and Sm populations. In the Ctr cytoplasm, CNOT1 pattern indicates granule formation, whereas in the Sm population these foci were dissolved, most likely as part of the rescue mechanism. (c) Finally, RPB1 in the control vessels exhibits cytoplasmic localization, whereas in the Sm samples RPB1 gets translocated to the nucleoplasm, revealing increased polymerase assembly and enhanced transcription, most likely as part of a compensatory attempt.

PARP-1 is best known as one of the signaling factors for DNA damage, which depends on the severity of stress attack and governs cells to specific fates [24]. Therefore, localization of PARP-1 is linked primarily to nucleoplasm. However, the fact is that very little is known about its tissue and cellular distribution. A detailed study on the PARP-1 expression pattern in cynomolgus monkey organs was recently published by Ferreira and coworker [25], which greatly expanded our knowledge regarding its tissue and subcellular localization (nuclear and/or cytoplasmic). They reported that in the blood vessels of many organs (thyroid, salivary glands, lung, ovary, uterus), the cytoplasmic localization of PARP-1 dominated. Consequently, the cytoplasmic appearance of PARP-1 we experienced in the UC blood vessels is not unprecedented under normal/physiological conditions.

Increased stress conditions, such as oxidative or nitrosative stress resulting from the accumulation of free radicals, trigger the expression and activation of PARP-1. In the Sm samples, venous endothelial cells responded with increased synthesis of PARP-1 to continuous stress stimuli. However, the heightened enzyme capacity remained partly unutilized, as a significant portion of PARP-1 translocated into the nucleus and became fragmented due to elevated levels of active Casp3. Consequently, a substantial fraction of PARP-1 in the Sm samples became unable to participate in the repair mechanism, and the truncated cPARP-1 migrated back to the cytoplasm. This inactivation hypothesis under stress conditions seems supported by the cPARP-1 pattern observed in the venous endothelium of the Ctr samples after ex vivo acute stress stimuli. Due to the low level of active Casp3 in the control venous endothelium, PARP-1 accumulated in the cell nucleoplasm, enabling it to organize various DNA repair processes when needed. During a short exposure of the Ctr vessels to heavy metal, we observed massive fragmentation of PARP-1 in the nucleus during the 30-min Cd^2 +^ -free recovery period, providing further support for our in vivo findings. On the other hand, the partial inactivation of PARP-1 by fragmentation could be beneficial for the Sm venous cell population, as increased activation of PARP-1 may contribute to the pathogenesis of various cardiovascular diseases.

Considering that PARP-1 is recognized as an essential co-factor in gene expression regulation by modulating the binding of transcription factors to specific promoter regions, the lack or inhibition of it under pathological conditions could also exert a protective effect on cell survival, maintenance of tissue homeostasis, or preservation of extracellular matrix integrity [26,27]. This protective effect has been documented in several referred studies [28,29].

In the case of CNOT1 labeling, the formation of distinct foci suggests an association between CNOT1 and membraneless organelles, particularly assemblysomes, in the control samples. Assemblysomes, which house ribosome nascent chain complexes stalled in the translation of stress-responsive genes and are formed by phase separation, are typically present in the cytoplasm of steady-state cells. This is in contrast to stress granules and p-bodies, which form in response to stress. Assemblysomes, upon genotoxic or proteotoxic stress, can exit their phase-separated state, simultaneously alleviating translation arrest from stress-responsive proteins and contributing to a timely stress response [20,30,31].

**Figure 7:**
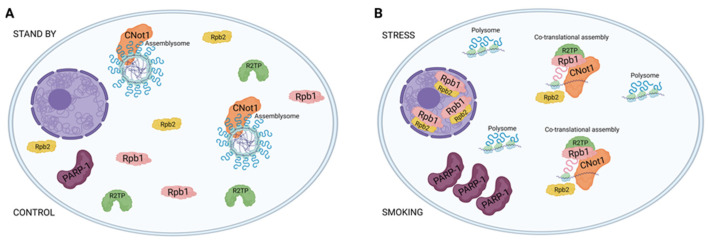
Stress induces changes in the distribution of CNOT1 within the cytoplasm, which impacts the assembly of RNA polymerase II and its subsequent transport into the nucleus, leading to increased levels of PARP-1 (created with Biorender.com). (**A**): Under normal circumstances, CNOT1 typically exhibits a granular pattern within the cytoplasm. The assembly of Rpb1 and Rpb2, crucial for their translocation into the nucleus, depends on CNOT1. However, the granular association of CNOT1, likely associated with assemblysomes, results in limited accumulation of Rpb1 in the nucleus. Consequently, transcription and subsequent expression of proteins such as PARP-1 remain low. (**B**): When exposed to stress, CNOT1 demonstrates a more dispersed cytoplasmic staining, suggesting a shift from assemblysomes to polysomes. This transition increases the availability of soluble CNOT1 for the assembly of Rpb1 and Rpb2, facilitating their nuclear accumulation. As a result, the relocation of RPB1 to the nucleus enhances overall transcription, leading to elevated levels of PARP-1 proteins.

Therefore, it is crucial for cells, especially stress-prone ones like blood-vessel-derived endothelial cells, to maintain a pool of assemblysomes. Despite CNOT1’s involvement in various processes, such as chaperoning activities of RPB1, a significant amount of CNOT1 can be sequestered to assemblysomes and stress granules [30,32]. Notably, in Sm samples, RPB1 becomes nuclear compared to control samples where RPB1 is predominantly cytoplasmic. We propose that in control samples, CNOT1 is present in cytoplasmic condensates, likely with assemblysomes, keeping it away from RPB1-RPB2 assembly processes. The RPB1 level already in the nucleus under steady-state conditions is sufficient to maintain gene expression homeostasis. Upon stress, assemblysomes transition from their phase-separated state, releasing CNOT1 and making it more accessible for RPB1 and RPB2 assembly processes. Consequently, more RNA polymerase II can enter the nucleus, leading to enhanced global transcription, as evidenced by elevated protein levels of PARP-1 proteins measured by mean fluorescence intensity in Sm samples compared to control (Figure 7). This observation is particularly striking in Sm veins concerning RPB1 and CNOT1. Umbilical cord vein endothelial cells directly encounter compounds from maternal smoking, as these vessels supply blood to the fetus. The difference in Sm UC veins and arteries regarding RPB1 and CNOT1 expression raises awareness of the damage the developing fetus faces in response to toxic materials from maternal cigarette smoke.

## 4. Materials and Methods

### 4.1. Human Samples

UC samples were obtained from the Department of Obstetrics and Gynecology at the University of Szeged, Hungary. The collection adhered to the principles outlined in the Declaration of Helsinki, and our study protocol received approval from the Ethics Committee of the Department of Obstetrics and Gynecology (16/2016). The study included UC fragments from neonates born to heavy-smoking mothers (at least 10 cigarettes per day throughout the full-term pregnancy) (n_Sm_ = 6) and age-matched neonates born to nonsmoking mothers (n_Ctr_ = 8). Clinical parameters of the study groups are summarized in Supplementary Table S1. The study protocol clearly stated definite exclusion criteria, including maternal age below 18 years, gestational age less than 37 weeks, infections, inflammatory conditions, gestational diabetes, high blood pressure, stroke, atherosclerosis, heart failure, drug treatment, intrauterine distress, malformations, or evidence of genetic disorders. The UCs were promptly transported to the laboratory after delivery for further processing. Age limit for the mothers was not considered below 18 years, minimum gestational age must be more than 37 weeks, no signs and symptoms of infections, inflammatory conditions, high blood pressure, stroke, gestational diabetes, atherosclerosis, and heart failure were taken into account as per the study protocol. Moreover, any type of clinical history related to drug treatment, intrauterine complications, malformations, or any evidence of congenital anomalies were also forbidden. After the delivery, the UCs were promptly transported to the laboratory for further processing.

### 4.2. Immunohistochemistry on the Cryopreserved UC Sections

Small UC pieces were fixed in 4% (*w*/*v*) paraformaldehyde in 0.05 M phosphate-buffer (PB), cryoprotected with 30% (*w*/*v*) sucrose in PB supplemented with 0.1% (*w*/*v*) Na-azide, embedded in Tissue-Tek^®^ O.C.T.™ obtained from Sakura Finetek Europe (Alphen aan den Rijn, The Netherlands), cryo-sectioned (16 µm), and mounted on Superfrost™ ultraplus^®^ microscope slides from Thermo Scientific (Waltham, MA, USA). The immunolabeling process involved permeabilization with 0.1% (*v*/*v*) Triton X-100 in PB, followed by blocking the nonspecific antibody binding sites with 4% (*w*/*v*) bovine serum albumin and 5% (*v*/*v*) normal goat serum in PB. For immunolabeling, primary antibodies were diluted in a ratio of 1:100, and the sections were incubated overnight at 4 °C. Primary antibodies: rabbit anti-PARP-1 monoclonal (C.384.8): MA5-15031; Invitrogen (Waltham, MA, USA), mouse anti-cleaved PARP-1 monoclonal (194C1439): sc-56196; Santa Cruz Biotechnology (Dallas, TX, USA), rabbit anti-CNOT1 polyclonal: ab234642; Abcam (Cambridge, UK), mouse anti-Pol II (RPB1) monoclonal (A-10): sc-17798 Santa Cruz Biotechnology (Dallas, TX, USA), rabbit, anti-cleaved Casp3 polyclonal: ab2302; Abcam (Cambridge, UK). Secondary antibodies were diluted in to 1:1000 and used alone or in combination in case of double labelling: goat anti-mouse or anti-rabbit IgG H & L conjugated with Alexa Fluor^®^ 488 or 647 (ab150077; ab150113; ab150115; ab150079, respectively Abcam (Cambridge, UK) for 2 h in room temperature. Cell nuclei were identified by counterstaining with 1 µg/mL 4′, 6-diamidino-2-phenylindole (DAPI) (D9542) from Sigma-Aldrich (St. Louis, MO, USA). Sections were mounted in Antifading BrightMount/Plus aqueous mounting medium: ab103748 Abcam (Cambridge, UK) for confocal microscopic analysis.

### 4.3. Ex Vivo Cd^2+^ Treatment on UC Pieces

UC fragments from both smoking and nonsmoking groups were dissected on ice within 1 h after delivery and divided into small pieces. To simulate acute exposure to heavy metals, the fragments were placed in 12-well cell culture plates containing Dulbecco’s Modified Eagle Medium (DMEM) with high glucose and with L-glutamine, DMEM, Lonza Bioscience, Basel, Switzerland: 12-604F) with and without 0.5 ng/µL Cd^2+^ in the form of cadmium acetate dihydrate (Sigma–Aldrich, St. Louis, MO, USA: 289159). Cd^2+^ exposure lasted for 30 min in a humidified incubator at 37 °C and 5% CO_2_. This was followed by rinsing twice with Cd^2+^-free DMEM and further incubation for 30 min, as a recovery phase. Finally, after completion of the incubation, UC fragments were fixed and prepared for embedding.

### 4.4. Confocal Microscopy Image Analysis

Sections were examined using a confocal laser scanning microscope, ZEISS LSM 880 equipped with Axiocam 503 mono (Carl Zeiss Microscopy GmbH, Germany). Image acquisition was performed with Zen 2.1 (black) software. Imaging of arteries and veins of each sample were captured in at least 5 independent fields of view. For antibody-labeled sections, with the following parameters excitation with Argon (488 nm) laser line for Alexa 488 fluorophore, Helium-Neon (633 nm) laser line for Alexa 647 fluorophore, violet (405 nm) laser line for DAPI, with detection filters in the range: 493–591 nm, 630–755 nm and 410–479 nm; pinhole = 1 airy unit. Eight-bit images were semiquantified with ImageJ 1.50i software (NIH, Bethesda, MD, USA). Visualization of sections were done using a confocal laser scanning microscope, ZEISS LSM 880 with Axiocam 503 mono (Carl Zeiss Microscopy GmbH, Germany). Capture of images was done with Zen 2.1 (black) software. For each sample (including both artery and vein), image acquisition was done from at least 5 independent fields of view or region of interest (ROI). Immunolabeling detection was done with the following protocol parameters: excitation with Argon (488 nm) laser line for Alexa 488 fluorophore, Helium-Neon (633 nm) laser line for Alexa 647 fluorophore, and the violet (405 nm) laser line for DAPI, having the detection filters in the range: 493 nm–591 nm, 630 nm–755 nm and 410 nm–479 nm; pinhole = 1 airy unit. Eight-bit images were semiquantified with ImageJ 1.50i software (NIH, USA).

To summarize the protocol, images were split into channels corresponding to specific antibody labeling, and endothelial regions were marked using the freehand selection tool. The fluorescent signals were evaluated with particle analysis with a running prerecorded command line. Optimization of this macro was done as per our study protocol. As a result, the region of interest (ROI) was saved and the mean gray values (as proportional to the fluorescent intensities) were measured and corrected to the background intensity. Background fluorescence was corrected by quantifying five independent, same-sized nonspecific fluorescent areas on each image. Data were summarized in MS^®^ Excel, and the fold of change in mean fluorescence intensity (MFI) were expressed, normalized to their own control values (~1).

### 4.5. Statistical Analysis and Graphic Representation

Statistical analyses and graphs were generated using GraphPad Prism version 8.0.1 (GraphPad Software, La Jolla, CA, USA). The results are represented as mean ± SEM. Sample groups were compared using an unpaired *t*-test (two-tailed), followed by a Mann-Whitney post hoc test. The differences were considered significant if *p* ≤ 0.05.

## 5. Conclusions

Maternal-smoking-induced alterations in the endothelial cells of umbilical cord vessels reveals a pivotal role of PARP-1 in regulating gene expression, particularly with response to stress. The observed significant impact of stress exposure levels on PARP-1 function emphasizes its crucial role in maintaining gene expression homeostasis and its potential implications for fetal health. Significantly, our findings suggest a potential novel regulatory mechanism under stress, wherein PARP-1 undergoes cleavage to prevent its involvement in several number of biological processes. This process, when unchecked, negatively impacts the extracellular matrix integrity of the blood vessel endothelial cells. Furthermore, the research unveils intriguing insights into the normal appearance of CNOT1 in condensates, likely within assemblysomes, as they exhibit dynamic behavior by dispersing in the cytoplasm in response to stress. The dissolution of CNOT1 foci in smoking-exposed samples suggests a potential rescue mechanism, shedding light on the intricate interplay between CNOT1 and cellular stress responses in the context of maternal smoking.

## Data Availability

Data supporting the reported results are available upon request.

## Supplementary Materials

The following supporting information can be downloaded at: https://www.mdpi.com/article/10.3390/ijms25073895/s1.
