# Supplementary material for: Stress-Induced Changes in Nucleocytoplasmic Localization of Crucial Factors in Gene Expression Regulation
Source: Int J Mol Sci. 2024 Mar 31;25(7):3895. doi: 10.3390/ijms25073895 (PMC11012061; doi:10.3390/ijms25073895)
Supplement: Supplementary file 1 [file ijms-25-03895-s001.zip › ijms-2897536-supplementary.pdf]

Stress induced changes in nucleocytoplasmic localization of crucial factors in gene expression regulation

Ali Khamit[1,a], Payal Chakraborty[1,a,†], Szabolcs Zahorán[1,††], Zoltán Villányi[1], Hajnalka Orvos[2] and Edit Hermesz [1,*]

[1] Department of Biochemistry and Molecular Biology, Faculty of Science and Informatics, University of Szeged, H-6701 Szeged, Hungary

[2]Department of Obstetrics and Gynecology, Albert Szent-Györgyi Medical School, University of Szeged, H-6701 Szeged, Hungary

* Correspondence: hermesz@bio.u-szeged.hu; Tel.: +36-(62)-544-887

[a] Contributed equally to this work.

[†] Present address: Department of Pharmacology, Calcutta Institute of Pharmaceutical Technology and Allied Health Sciences, Uluberia, Howrah -711316, West Bengal, India

[††] Present address: Department of Cell Physiology and Metabolism, University of Geneva, 1211 Geneva 4, Switzerland

Table S1. The clinical parameters of the study groups and the maternal age		
**Clinical Parameters**	**Ctr sample population**	**Sm sample population**
**Numbers of Samples (N)**	n= 14	n = 12
**Gestational age (weeks)**	38.94±1.1	38.03 ± 0.67
**Birth weight (kg)**	3.464 ± 0.46	2.909 ± 0.618(***)
**APGAR score at 1 min**	9.36 ± 0.36	8.88 ± 0.66 (*)
**25-50 Percentil frequency(%)**	17.72	44.59 (**)
**Chest circumference (cm)**	33.1±1.84	30.94±2.7(****)
**Head circumference (cm)**	34.49±1.44	32.50±2.19(***)
**Blood sample pH**	7.27 ± 0.07	7.25 ± 0.12
**Maternal age (years)**	30.52 ± 3.34	32.7 ± 4.73

Statistical analysis was done by using unpaired t-test (two-tailed) and Mann-Whitney post-hoc test to compare ranks. (*$p \leq 0.05$, **$p \leq 0.01$, ***$p \leq 0.001$, ****$p \leq 0.0001$**** $p \leq 0.0001$)

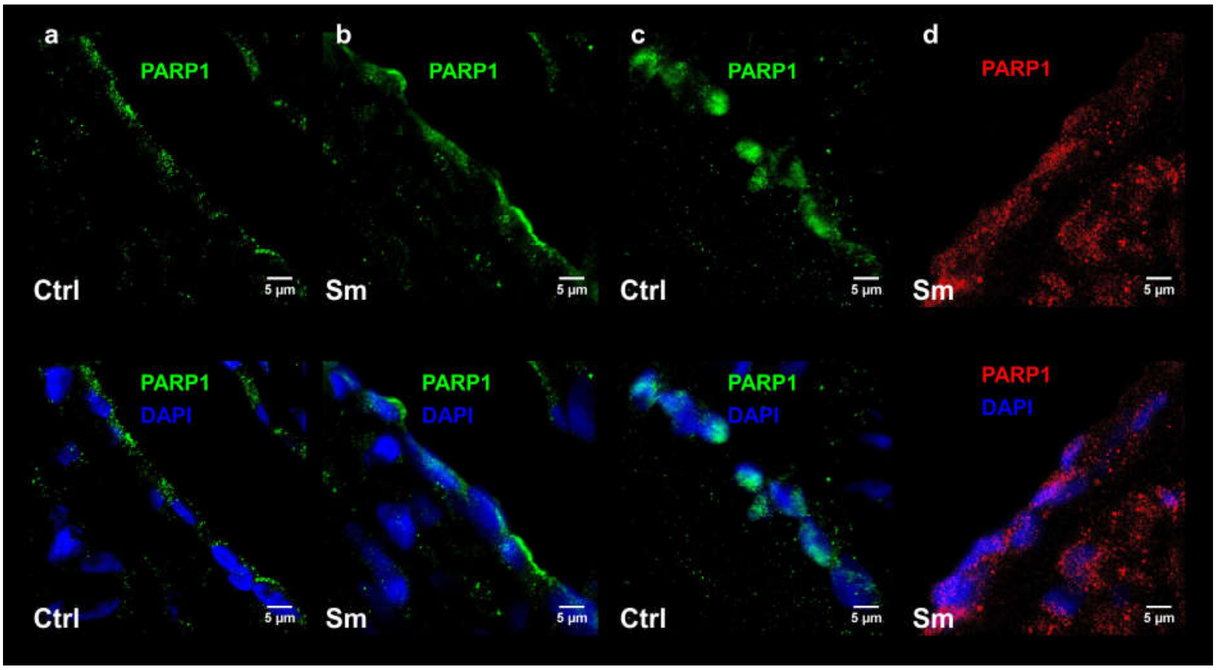

Figure S1. Representative confocal images showing PARP-1 pattern.

PARP-1 localization was followed in the Ctr (**a** and **c**) and Sm (**b** and **d**) UC vessels. Arteries (**a** and **b**) and veins (**c** and **d**) were immunolabeled with monoclonal rabbit anti-PARP-1 antibody with 1:100 dilutions, followed by Alexa 488 (green) or Alexa 647 (red) anti-rabbit secondary antibodies labelling with dilution 1:1000. Cell nuclei were counterstained with 1µg/mL 4', 6-diamidino-2-phenylindole (blue). Slides were mounted and examined using a confocal laser scanning microscope, ZEISS LSM 880 equipped with Axiocam 503 mono.

A

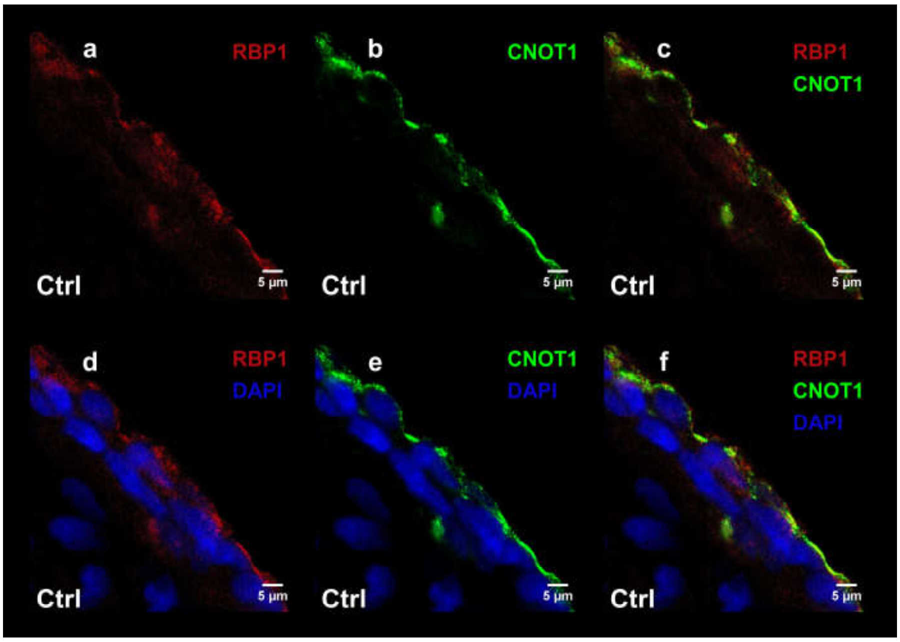

B

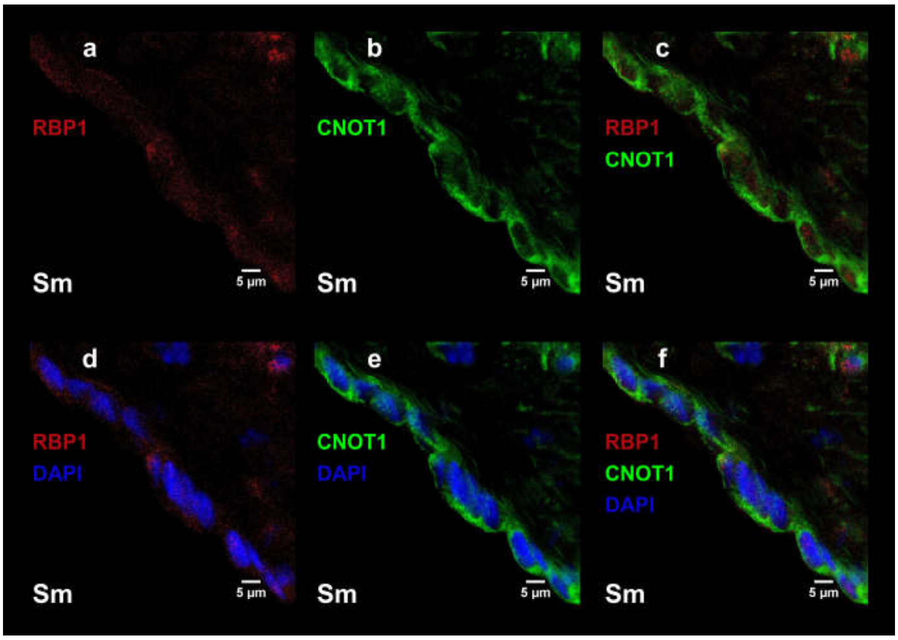

Figure S2. Representative confocal images showing RPB1 and CNOT1 localization in the cytoplasm and nucleoplasm of the endothelial cells in the Ctr (**A**) and Sm (**B**) arteries.

Expression pattern of RPB1 and CNOT1 was followed in Ctr (**A**) and Sm (**B**) arteries. Double immunolabeling were carried out with mouse monoclonal anti-RPB1 (**a** and **d**) and rabbit polyclonal anti-CNOT1 (**b** and **e**) antibodies (1:100) followed by Alexa 647 (red) and Alexa 488 (green) anti-mouse/rabbit secondary antibodies labelling (1:1000). Cell nuclei were counterstained with 1µg/mL 4', 6-diamidino-2-phenylindole (blue). Panels (**c** and **f**) showed the merged images. Slides were mounted and examined using a confocal laser scanning microscope, ZEISS LSM 880 equipped with Axiocam 503 mono.

A

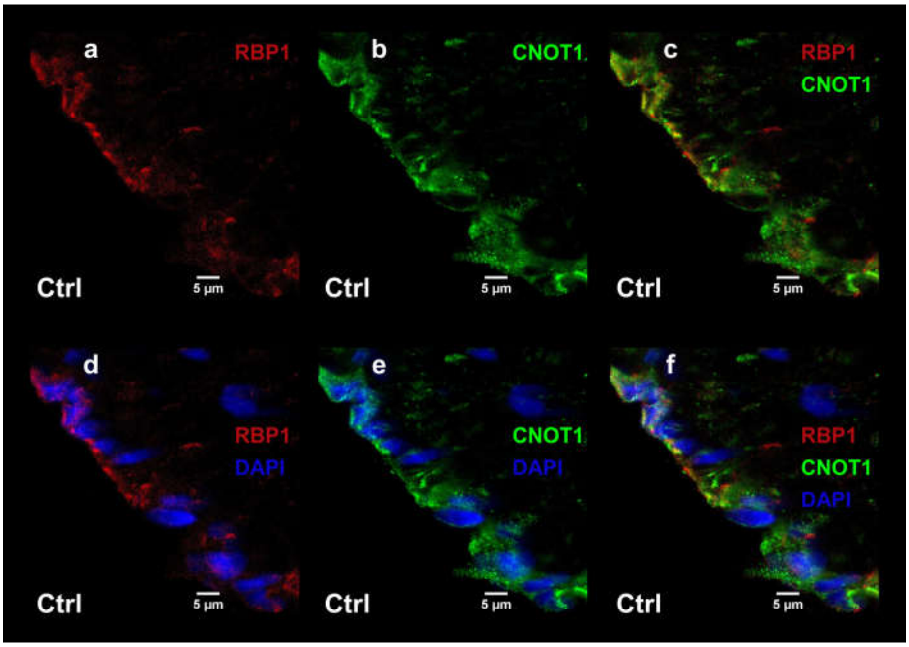

B

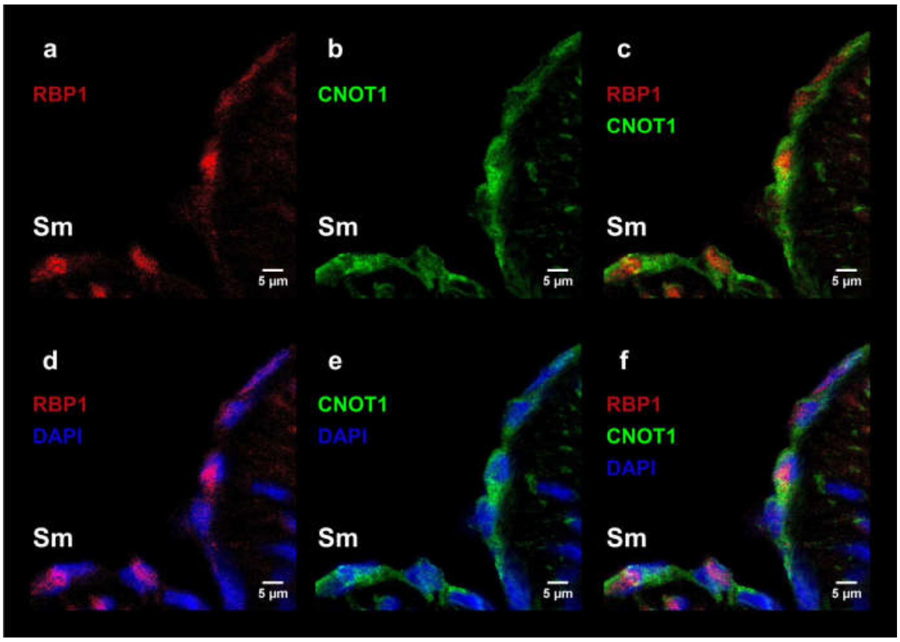

Figure S3. Representative confocal images presenting RPB1 and CNOT1 localization to the cytoplasm and nucleus of the endothelial cells in the Ctr (**A**) and Sm (**B**) veins.

Double immunolabeling were carried out with mouse monoclonal anti-RPB1 (**a** and **d**) and rabbit polyclonal anti-CNOT1 (**b** and **e**) antibodies (1:100) followed by Alexa 647 (red) and Alexa 488 (green) anti-mouse/rabbit secondary antibodies labelling (1:1000). Cell nuclei were counterstained with 1µg/mL 4', 6-diamidino-2-phenylindole (blue). Panels (**c** and **f**) showed the merged images. Slides were mounted and examined using a confocal laser scanning microscope, ZEISS LSM 880 equipped with Axiocam 503 mono.
